# Molecular and Biochemical Characterization of a Bimodular Xylanase From *Marinifilaceae* Bacterium Strain SPP2

**DOI:** 10.3389/fmicb.2019.01507

**Published:** 2019-07-02

**Authors:** Zhenggang Han, Fang Shang-guan, Jiangke Yang

**Affiliations:** College of Biology and Pharmaceutical Engineering, Wuhan Polytechnic University, Wuhan, China

**Keywords:** Fn3 domain, glycoside hydrolase, *Marinifilaceae* bacterium, xylanase, xylooligosaccharide

## Abstract

In this study, the first xylantic enzyme from the family *Marinifilaceae*, XynSPP2, was identified from *Marinifilaceae* bacterium strain SPP2. Amino acid sequence analysis revealed that XynSPP2 is a rare Fn3-fused xylanase, consisting of a signal peptide, a fibronectin type-III domain (Fn3), and a C-terminal catalytic domain belonging to glycoside hydrolase family 10 (GH10). The catalytic domain shared 17–46% identities to those of biochemically characterized GH10 xylanases. Structural analysis revealed that the conserved asparagine and glutamine at the glycone −2/−3 subsite of GH10 xylanases are substituted by a tryptophan and a serine, respectively, in XynSPP2. Full-length XynSPP2 and its Fn3-deleted variant (XynSPP2ΔFn3) were overexpressed in *Escherichia coli* and purified by Ni-affinity chromatography. The optimum temperature and pH for both recombinant enzymes were 50°C and 6, respectively. The enzymes were stable under alkaline condition and at temperature lower than 50°C. With beechwood xylan as the substrate, XynSPP2 showed 2.8 times the catalytic efficiency of XynSPP2ΔFn3, indicating that the Fn3 module promotes xylanase activity. XynSPP2 was active toward xylooligosaccharides (XOSs) longer than xylotriose. Such a substrate preference can be explained by the unique −2/−3 subsite composition in the enzyme which provides new insight into subsite interaction within the GH10 family. XynSPP2 hydrolyzed beechwood xylan into small XOSs (xylotriose and xylotetraose as major products). No monosaccharide was detected by thin-layer chromatography which may be ascribed to putative transxylosylation activity of XynSPP2. Preferring long XOS substrate and lack of monosaccharide production suggest its potential in probiotic XOS manufacture.

## Introduction

Plant material mainly consists of cellulose, hemicellulose, and lignin (Tuck et al., 2012). Xylan, a β-1,4-linked xylopyranose polymer, is the most common constituent of hemicellulosic polysaccharide in the biosphere (Linares-Pasten et al., 2018). Intensive research focused on developing different innovative technologies to exploit xylan (Van Dyk and Pletschke, 2012). Enzymatic decomposition is attractive because it is environmentally friendly. Xylan biodegradation is carried out by many hydrolytic enzymes. Among them, the enzymes that randomly break down the internal glycosidic bond in the linear xylan main chain are termed endo-β-1,4-xylanases (EC. 3.2.1.8, normally referred to as xylanases) (Nguyen et al., 2018).

Xylanases from diverse groups of microorganisms have attracted increasing attention in the last few decades because of their enormous biotechnological potential in a wide variety of industrial processes (Linares-Pasten et al., 2018). Xylanases have been applied in lignocellulose materials saccharification (preparing fermentable sugars) (Basit et al., 2018a), animal feeds (as additives) (Ndou et al., 2015), chlorine-free bleaching of wood pulp (Thomas et al., 2015), and brewer’s spent grain saccharification (Amor et al., 2015). In addition, the production of xylooligosaccharides (XOSs) as putative prebiotics using xylanases has obtained increasing interest (Morgan et al., 2017; Linares-Pasten et al., 2018; Nordberg Karlsson et al., 2018). XOSs with a degree of polymerization between 2 and 10 units demonstrate great prebiotic effects and are potential ingredients in food and pharmaceutical preparations (Aachary and Prapulla, 2011; Nieto-Domínguez et al., 2017).

In the Carbohydrate Active Enzymes database (CAZY^1^), the most characterized xylanases are mainly grouped into glycoside hydrolase families 10 and 11 (GH10 and GH11) according to the amino acid sequence homologies of their catalytic domains (Lombard et al., 2014; Nguyen et al., 2018). Compared with GH11 xylanases, GH10 xylanases have a broader substrate specificity. In addition, they are active on the decorated heteroxylans to some extent, producing smaller enzymatic products than GH11 xylanases (Pollet et al., 2010). GH10 xylanases exhibit an (β/α)_8_ barrel structure that folds into a bowl shape. A set of xylose-binding subsites arranged on the outside surface of xylanases determines the position-specific binding and cleavage of a substrate. The glycosidic bond linking the xylose residues at the −1 and +1 subsites is cleaved. In general, the subsites at the glycone region are well conserved and strong in xylose residue binding, whereas the subsites at the aglycone region are less conserved and weak in xylose residue binding.

Different from GH11 xylanase, GH10 xylanases generally show higher activity on small XOS molecules (including xylotriose) and produce XOS with lower degree of polymerization (X2–X5) and xylose (Linares-Pasten et al., 2018). Being active toward small XOSs and producing of monosaccharide to some extent are disadvantages for specific application of xylanase, such as probiotic XOS manufacture. Structural study has shown that the shortest hydrolysable substrate for catalysis is determined by subsite interactions at the glycone region of substrate-binding cleft (Schmidt et al., 1999). A small number of amino acid variations in the substrate-binding cleft of a subset of GH10 xylanases, mostly the −2/−3 region (distal amino acids constituting the −2 subsite), confers subtle differences in substrate specificity and cleavage pattern to the enzymes compared with the other GH10 xylanases (Andrews et al., 2000; Pell et al., 2004). For example, a Glu/Gly substitution at the −2/−3 subsite of *Cj*Xyn10C from *Cellvibrio japonicus* alerts the affinity for XOSs but does not impair the affinity for long substrates (xylan); in addition, a tyrosine insertion at the −2/−3 subsite of *Cj*Xyn10C changes the cleavage pattern of xylotetraose from “−2 to +2” to “−3 to +1” (Pell et al., 2004). Accordingly, to screen the xylanases harboring unique variations within the substrate-binding cleft is an effective way to characterize xylanases with changed substrate preference and product pattern.

Current advances in microbial genome or metagenome sequencing provide opportunities to identify a large number of xylanases with novel sequences (Basit et al., 2018b). Enzymes from marine microorganisms have attracted considerable attention because they are possibly unique in primary sequence and biochemical property (Kennedy et al., 2008; Trincone, 2011). Therefore, we provided special attention to uncharacterized xylanases in the genome of marine microorganisms. A putative GH10 xylanase, XynSPP2 from *Marinifilaceae* bacterium strain SPP2, was recently isolated from the Antarctic marine sediment (Watanabe et al., 2018). The xylanase particularly attracted our attention due to its unique architecture at glycone subsites as revealed by multiple amino acid sequence alignment and 3-D structure modeling. The present paper reports the bioinformatic and biochemical characterization of XynSPP2. It is also the first xylantic enzyme from the family *Marinifilaceae*. Amino acid sequence analysis suggested that XynSPP2 consists of a fibronectin type 3 (Fn3) domain and a GH10 catalytic domain. Recombinant full-length XynSPP2 and GH10 domain (XynSPP2ΔFn3) were produced by *Escherichia coli* system. Xylanase assay showed that the Fn3 domain contributes to the catalytic efficiency of XynSPP2. Thin-layer chromatography (TLC) indicated recombinant XynSPP2 displayed very weak activity toward xylotriose and preferred cleaving xylotetraose in a “−3, +1” mode. Such a hydrolytic property is different from the general characteristic of GH10 xylanases. Structural analysis suggested unique −2/-3 subsite in XynSPP2 may account for its unique hydrolytic property which provides new insight into subsite interaction of GH10 xylanase. In addition, no monosaccharide was detected by TLC assay. All of these properties suggest that XynSPP2 is suitable for XOS production.

## Materials and Methods

### Materials

Nucleotide sequence encoding of XynSPP2 was obtained from the genome of *M.* bacterium strain SPP2 (GenBank accession number: NZ_AP018042.1). DNA fragments of XynSPP2 were synthesized by GENEWIZ Suzhou (Suzhou, China) after codon optimization (designed for recombinant protein production in *E. coli* using Codon OptimWiz software developed by GENEWIZ). The nucleotide sequence of codon-optimized *XynSPP2* has been deposited in GenBank under the accession number of MK722389. Beechwood xylan and XOSs (xylobiose, xylotriose, xylotetraose, xylopentaose, and xylohexaose) were purchased from Megazyme Corp (Wicklow, Ireland). Molecular mass standards were purchased from Bio-Rad Laboratories (Shanghai, China).

### Amino Acid Sequence Analysis and Structure Homology Modeling

**FIGURE 1:**
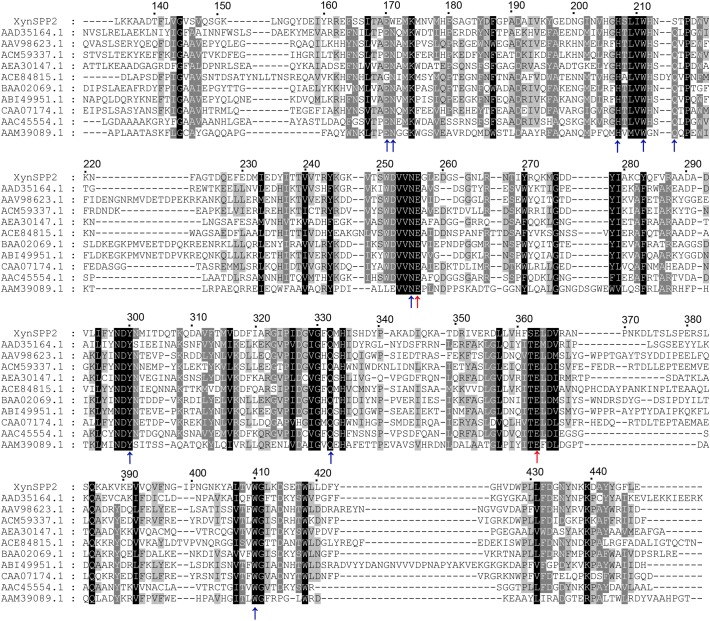
Multiple sequence alignment of GH10 domains of XynSPP2 and representative xylanases. Amino acid sequences were aligned using Clustal Omega. Strictly conserved and similar amino acids are shown in black and gray background, respectively. The numbers of amino acids refer to XynSPP2. The GH10 domain sequences are represented by GenBank accession numbers of their intact proteins, with AAD35164.1 for xylanase from *Thermotoga maritima* MSB8 (TmxB), AAV98623.1 for xylanase from *Bacillus halodurans*, ACM59337.1 for xylanase from *Caldicellulosiruptor bescii* DSM 6725 (*Cb*Xyn10B), AEA30147.1 for xylanase from *Cellulomonas fimi* ATCC 484 (*Cf*Xyn10A), ACE84815.1 for xylanase from *Cellvibrio japonicus* Ueda107 (*Cj*Xyn10C), BAA02069.1 for xylanase from *Clostridium stercorarium*; ABI49951.1 for xylanase from *Geobacillus stearothermophilus* (XT6), CAA07174.1 for xylanase from *Paenibacillus barcinonensis* (*Pb*Xyn10B), AAC45554.1 for xylanase from *Streptomyces halstedii*, AAM39089.1 for xylanase from *Xanthomonas citri*, and AYC81220.1 for xylanase from uncultured bacterium. Red and blue arrows highlight the putative catalytic amino acids (acid/base catalyst and nucleophile) and amino acid residues involved in xylose residue binding, respectively.

Signal peptide and functional domain annotation was performed using SignalP-5.0^2^ (Almagro Armenteros et al., 2019) and Conserved Domain Search^3^ (Marchler-Bauer et al., 2017), respectively. GenBank database search for XynSPP2 sequence (GenBank accession number: WP_096428726.1) analysis was carried out using BLASTp^4^. Amino acid sequence (full-length enzymes or their GH10 domains) comparison between XynSPP2 and characterized GH10 xylanases to date (320 sequences were used)^5^ was conducted using Clustal Omega^6^ (Sievers et al., 2011). A phylogenetic tree of the GH10 domains of the characterized xylanases was constructed using MEGA 7 (Kumar et al., 2016). Conserved amino acid residues in the active-site cleft of the GH10 xylanases was demonstrated using WebLogo (Crooks et al., 2004) on the basis of the result of the GH10 domain alignment. An alignment of GH10 domains of 10 representative xylanases [all of their X-ray structures are available in Protein Data Bank (PDB)] was performed to prepare an alignment figure (Figure 1). A 3-D structural model of XynSPP2 was built by homology modeling using SWISS-MODEL^7^ (Waterhouse et al., 2018). The quality of the resulting model was evaluated by MolProbity (Chen et al., 2010). Structural graphics were prepared using PyMOL (Schrödinger LLC, Cambridge, MA, United States).

### Recombinant XynSPP2 Production and Purification

*E. coli* DH5α (Thermo Fisher Scientific, Shanghai, China) was used for cloning. Full-length XynSPP2 and catalytic domain (XynSPP2ΔFn3) were subcloned into the expression vector pET-28a (Novagen, San Diego, CA, United States). Overnight cultures of *E. coli* BL21 (DE3) cells (Thermo Fisher Scientific, Shanghai, China) harboring recombinant plasmids (pET-28a-XynSPP2 or pET-28a-XynSPP2ΔFn3) were prepared to inoculate 200 ml of ZYM 5052 autoinduction medium supplemented with 100 μg/ml kanamycin, and 34 μg/ml chloramphenicol in a 2 L shake flask (Studier, 2005). After 4-h incubation at 37°C and 250 rpm, the cultures were cooled and further incubated for 24 h at 20°C before harvested by centrifugation. The pellet was resuspended in sodium phosphate buffer (50 mM, pH 7), and the cells were broken by sonication. The cell-free extract was obtained by centrifugation at 25,000 × *g* for 30 min at 4°C. Protein purification was conducted by Ni-affinity chromatography as described previously (Han et al., 2018). The homogeneity of the recombinant protein was checked by sodium dodecyl sulfate–polyacrylamide gel electrophoresis (SDS-PAGE). The pure fractions were pooled and protein concentration was determined by Bradford using bovine serum albumin as the standard.

### Xylanase Activity Assays

Xylanase activities were measured with beechwood xylan as the substrate. Each reaction mixture contained 100 μl of beechwood xylan (10 mg/ml), 100 μl of diluted purified XynSPP2 or XynSPP2ΔFn3 (20 μg/ml), and 200 μl of citrate-phosphate buffer (200 mM, pH 6). The reactions were performed at 50°C for 10 min and stopped by adding 200 μl dinitrosalicylic acid. Xylanase activity was routinely quantified by determining the reducing sugar released from reactions with xylose as the standard (Bailey et al., 1992). Each assay was performed in triplicate. One unit of xylanase activity was defined as the amount of enzyme that is able to release 1 μmol xylose per minute under the assay condition.

### Effects of Temperature and pH on Xylanase Activity

Citrate-phosphate buffers (pH 3, 4, 5, 6, 7, and 8) and glycine-NaOH buffers (100 mM, pH 9, 10, and 11) were used to determine the pH profile of the recombinant xylanases. The optimum temperature was determined by measuring the xylanase activities at pH 6 for 10 min over the range from 10 to 70°C.

### Thermal Stability and pH Stability of Recombinant Xylanases

Thermal stability was evaluated by measuring the residual activity at the optimum conditions (50°C and pH 6) after incubating xylanase at different temperatures for 1 h. To analyze the stability at different pH values, the enzymes were incubated at pH 3-11 and 20°C for 1 h before subjecting them to the activity assay under optimal conditions. Purified enzymes were sufficiently diluted with buffers to ensure that the desired pH values were obtained at incubation and assay steps. In specific, for each assay, 0.5 μl of purified enzyme was at least 20 times diluted with buffers of different pH values for incubation, and the enzymes after incubation (about 20 μl) were subjected to xylanase assay (200 μl buffer of pH 6 was in the reaction mixture).

### Effects of Metal Ions, Chemical Reagents, and Salt Concentration on Xylanase Activity

The effects of different chemical reagents on xylanase activity were measured at 50°C and pH 6 for 10 min using purified XynSPP2. Each metal ion (prepared using CaCl_2_, CoCl_2_, FeSO_4_, MgSO_4_, ZnSO_4_, NiSO_4_, and MnCl_2_) was added at a final concentration of 5 mM. Then, 5% (glycerol, *n*-butanol, ethanol, methanol, isopropanol, and acetone) or 10% [acetone and dimethyl sulfoxide (DMSO)] of organic solvent was added individually to the reaction to study their effects on xylanase activity. The effects of ethylenediaminetetraacetic acid (EDTA) (10 mM), dithiothreitol (DTT) (10 mM), β-mercaptoethanol (β-ME) (0.5%), Triton X-100 (0.5%), sodium dodecyl sulfate (SDS) (10 mM), urea (100 mM), ammonium sulfate (100 mM), and guanidine hydrochloride (GuHCl) (100 mM) were also investigated. NaCl were added at a final concentration of 0.5–2.5 M to study the salt tolerance of XynSPP2.

### Kinetic Parameter Determination

The reactions to determine the values of maximum velocity (*V_max_*), Michaelis–Menten constant (*K_m_*), and turnover number (*k_cat_*) for XynSPP2 were performed at 50°C in citrate-phosphate buffer (pH 6) for 5 min. Beechwood xylan was used as the substrate at concentrations ranging from 0.5 to 6 mg/ml. *V_max_* and *K_m_* were determined using the Lineweaver–Burk method. The *k_cat_* value was calculated from the *V_max_* value according to the amount of enzyme used in the reaction.

### Hydrolytic Products Determination by Thin-Layer Chromatography

The hydrolytic products of XynSPP2 against XOSs were detected by TLC. The reaction mixtures included 0.5 μg of purified enzyme, and 20 μM substrates in citrate-phosphate buffer (pH 6.5). A 15-μl aliquot was taken at different time points and heated at 95°C for 10 min. The hydrolysate profiles were analyzed on a Silica Gel 60 TLC plate (Merck, Darmstadt, Germany) using a solution of *n*-butanol/acetic acid/water (10:5:1, v/v) as the solvent. Spots were visualized by spraying with staining solution [0.5 % sulfuric acid in methanol (v/v)] and heating at 115°C for 5 min. A mixture of XOSs (X1–X6) was used as the standard.

## Results

### Sequence and Structural Analysis of XynSPP2

*Marinifilaceae* bacterium strain SPP2 is a recently isolated polar microorganism classified into the family *Marinifilaceae* (Watanabe et al., 2018). Less commonly, as a Gram-negative bacterium, it encodes several predicted xylantic enzymes. XynSPP2 is one of the enzyme encoded by an open reading frame of 1353 bp (450 amino acid residues). The theoretical molecular mass and deduced isoelectric point of XynSPP2 are 50,461.40 Da and 4.87, respectively. A 24-residue signal peptide is present at the amino-terminus of protein as predicted by SignalP 5.0 (*D* = 0.712, *D*-cutoff = 0.570). The signal peptide is followed by an Fn3 domain and a GH10 domain as indicated by analysis using the Conserved Domain Search.

BLASTp search indicated that XynSPP2 was mostly identical (84%) to putative β-1,4-xylanase from *Labilibaculum filiforme* (GenBank accession number: WP_101261729.1), which has the same domain arrangement. The Fn3 and GH10 domains in XynSPP2 also shared the highest identities (73 and 91%) to those from *L. filiforme* xylanase, respectively. The second most identical sequence was hypothetical protein from *Saccharicrinis fermentans* (GenBank accession number: WP_044212766.1), which is 55% identical to XynSPP2. Amino acid sequence alignment showed that XynSPP2 was 17–46% identical to those characterized GH10 xylanases collected in the CAZy database (320 sequences of characterized GH10 xylanases). Among the characterized GH10 xylanases, XynSPP2 showed the highest sequence identity (46%) to the xylanase from *Flavobacterium johnsoniae* UW101 (GenBank accession number: ABQ06877.1). The GH10 domain of XynSPP2 was also mostly identical to that of *F. johnsoniae* UW101 xylanase, with 49% sequence identity (Supplementary Figure S1). Multiple sequence alignment also revealed the putative catalytic residues (acid/base catalyst: Glu256; nucleophile: Glu363) and amino acids involved in substrate binding (Glu170, Trp171, Lys174, His207, Ser214, Asn255, Trp300, Gln332, His334, and Trp410) in XynSPP2 (Figure 1 and Supplementary Figure S2). The latter were largely conserved in XynSPP2 except two variations at the −2/−3 subsite (Figure 1 and Supplementary Figure S2). The conserved asparagine and glutamine at −2/−3 in GH10 xylanases are replaced for tryptophan (Trp171) and serine (Ser214), respectively (Figure 1). These two natural variations are also present in the putative GH10 xylanase from *L. filiforme*.

**FIGURE 2:**
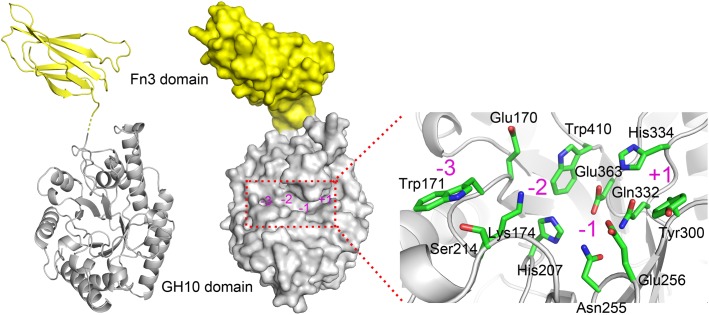
Predicted 3-D structure of XynSPP2. XynSPP2 consists of an N-terminal Fn3 domain (yellow) and a C-terminal GH10 domain (gray). Overall structural architecture of XynSPP2 is presented as cartoon (left) and surface (middle) models. Amino acids constituting the active-site cleft of XynSPP2 are shown in green stick (right). Subsites in the active-site cleft are indicated by magenta numbers.

Due to the absence of a model template for full-length XynSPP2 in PDB, the crystal structure of the GH10 domain of *Cj*Xyn10C (PDB entry: 1US3) and crystal structure of the Fn3 domain from human Contactin (PDB entry: 4N68) were used as the templates to generate model structures of the Fn3 and GH10 domains in XynSPP2, respectively. The amino acid similarity between the GH10 domains of XynSPP2 and *Cj*Xyn10C is 56% (35% identity), and the similarity between the Fn3 domains of XynSPP2 and human Contactin is 41% (24% identity). The qualities of both modeled structures were acceptable as indicated by the good QMEAN *Z*-score (Benkert et al., 2011) (2.18 for the GH10 domain and 3.83 for the Fn3 domain) from the SWISS-MODEL server pipeline and excellent structure validation statistics by MolProbity (MolProbity score: 1.81, 85^th^ percentile). All amino acids situated in the substrate-binding cleft showed a reliable geometry (allowable torsion angles and no steric problem). A structural model of intact XynSPP2 was obtained by assembling the two domains manually (Figure 2). The resulting structure indicated that the GH10 domain of XynSPP2 exhibits a typical (β/α)_8_-barrel of GH10 xylanases composed of alternating α-helices and β-strands. The Fn3 domain shows the conserved β-sandwich fold consisting of two beta sheets (one containing four strands and the other sheet containing three strands) (Figure 2). Trp171 and Ser214 comprise the distal edge of −2 subsite (−2/−3) (Figure 2), which agrees with the prediction from multiple amino acid sequence alignment (Figure 1).

### Expression and Purification of Recombinant XynSPP2 and XynSPP2ΔFn3

**FIGURE 3:**
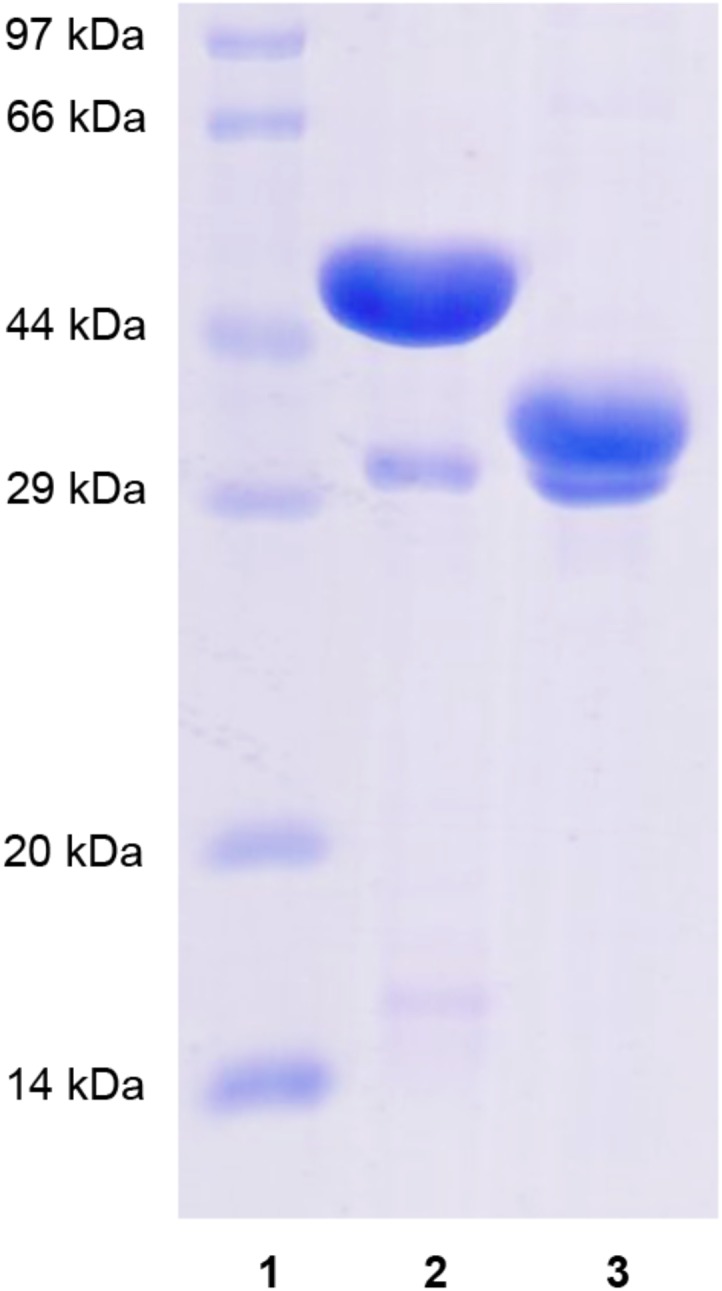
SDS-PAGE analysis of recombinant XynSPP2 and XynSPP2ΔFn3. Lane **(1)**, standard marker proteins; lane **(2)**, purified recombinant XynSPP2; lane **(3)**, purified recombinant XynSPP2ΔFn3.

Recombinant XynSPP2 (amino acid residues 25–450) and XynSPP2ΔFn3 (amino acid residues 137–450) were produced using the *E. coli* system and purified by Ni-affinity chromatography. Both recombinant proteins had a polypeptide of 34 amino acids from the pET-28a vector on their N-terminals and were purified to electrophoretic homogeneity (Figure 3). The purified XynSPP2 and XynSPP2ΔFn3 exhibited a single band with molecular masses that corresponded to their calculated values of 51,364.15 and 39,244.76 Da, as shown in SDS–PAGE (Figure 3).

### Biochemical Properties of XynSPP2 and XynSPP2ΔFn3

**FIGURE 4:**
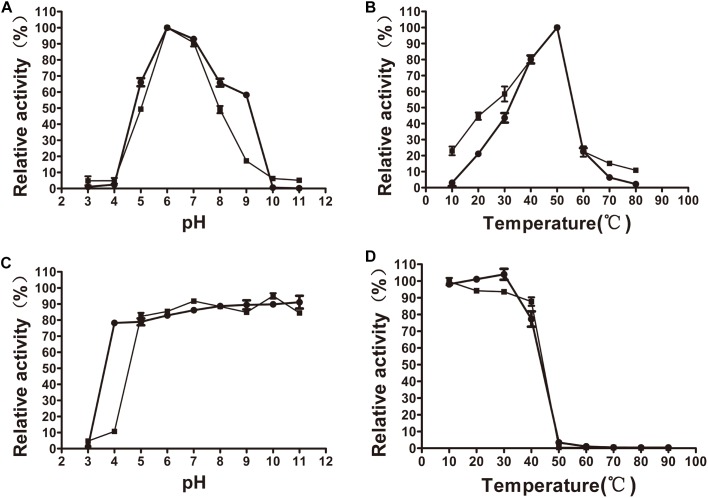
Effects of pH and temperature on the activity and stability of recombinant XynSPP2ΔFn3 (blank squares) and XynSPP2 (blank dots). **(A)** Optimal pH of XynSPP2ΔFn3 and XynSPP2. **(B)** Optimal temperatures of XynSPP2ΔFn3 and XynSPP2. **(C)** pH stability of XynSPP2ΔFn3 and XynSPP2. **(D)** Thermal stability of XynSPP2ΔFn3 and XynSPP2.

The xylanase activity of XynSPP2 or XynSPP2ΔFn3 was tested on β-1,4-linked xylose polysaccharide beechwood xylan. Both recombinant enzymes had similar pH and temperature profiles and exhibited a pH optima of 6 (Figure 4A) and temperature optima of 50°C (Figure 4B). XynSPP2 retained approximately 60% maximum activity over pH 5–9 and at least 40% maximum activity at 30°C–50°C. Compared with full-length XynSPP2, XynSPP2ΔFn3 was active across a narrower pH range (in particular of alkaline range) and a broader temperature range.

Both XynSPP2 and XynSPP2ΔFn3 were stable above pH 4, retaining more than 80% of the maximum activity (Figure 4C). Deletion of the Fn3 domain almost did not affect the thermal stability of XynSPP2, as demonstrated by the nearly complete loss of their activity after incubation at 50°C for 1 h (Figure 4D).

**FIGURE 5:**
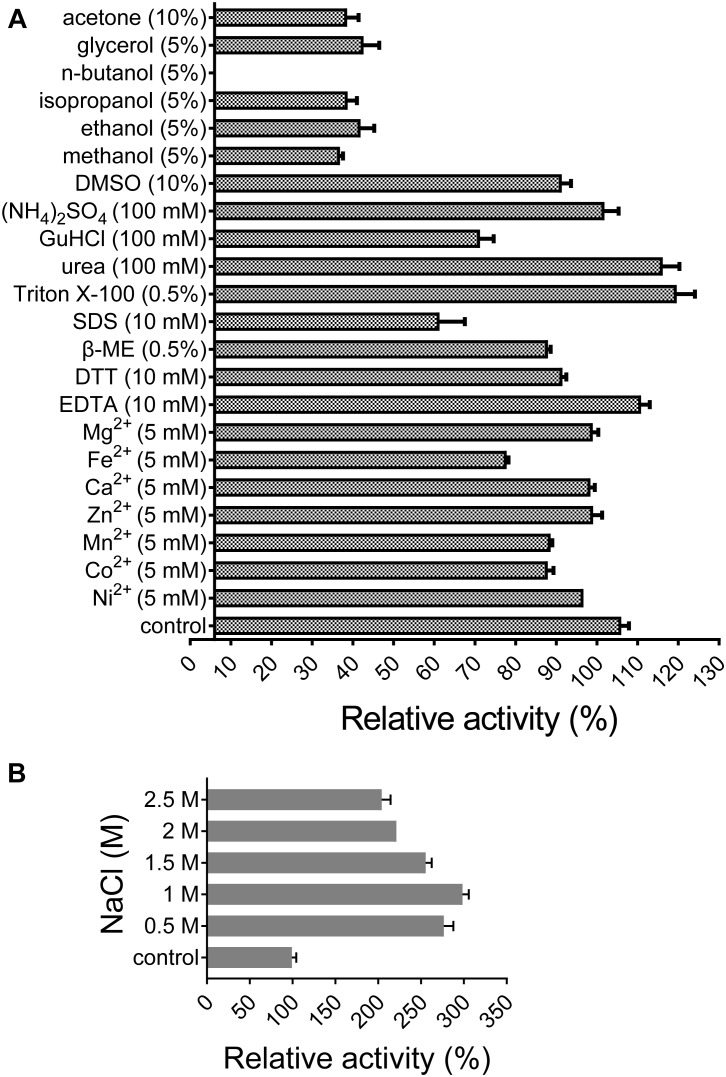
Effects of metal ions **(A)**, chemicals **(A)**, and NaCl **(B)** on the activity of recombinant XynSPP2.

All of the tested divalent cations (Co^2+^, Ca^2+^, Fe^2+^, Mg^2+^, Mn^2+^, Ni^2+^, and Zn^2+^) at 5 mM suppressed the activity of XynSPP2 by different degrees (ranging from 6 to 28%) relative to its original activity. Among them, the negative effect of Fe^2+^ was the most potent (Figure 5A). The activity of XynSPP2 increased by approximately 5% in the presence of EDTA. Reducing reagents, DTT (10 mM) and β-mercaptoethanol (10 mM), inhibited the activity of XynSPP2 by 15 and 20%, respectively. Anionic detergent SDS (10 mM, approximately 0.3%) and non-ionic surfactant Triton X-100 (0.5%) showed the reverse effect, retaining 55 and 113% xylanase activities, respectively. In the presence 5% of organic solvents, ethanol, methanol, isopropanol, and glycerol, XynSPP2 retained approximately 30% relative activity. The activity of XynSPP2 was completely inhibited in the presence of 5% *n*-butanol. The other tested organic solvents acetone and DMSO at 10% showed inhibitory effect. When the xylanase reactions were conducted in the presence of ammonium sulfate, urea, or GuHCl at a concentration of 100 mM, XynSPP2 showed 95, 110, and 65% of its original activity, respectively.

XynSPP2 was halophilic as demonstrated by greatly enhanced xylanase activity with the addition of a certain amount of NaCl in the reaction mixtures (Figure 5B). When the reactions were performed in the presence of NaCl at concentrations ranging from 0.5 to 2.5 M, XynSPP2 exhibited more than 210% of its original activity.

The whole enzyme XynSPP2 had a *K_m_* value of 0.97 mg/ml for beechwood xylan (Table 1 and Supplementary Figure S3). The absence of the Fn3 domain led to a *K_m_* value of 1.81 times that of intact XynSPP2. The *k_cat_* values of XynSPP2 and XynSPP2ΔFn3 were 178.19 and 117.27 s^−1^, respectively. The catalytic efficiency (as measured by *k_cat_/K_m_*) of XynSPP2ΔFn3 was approximately one third that of XynSPP2.

**Table 1:** Kinetic parameters of XynSPP2 and XynSPP2ΔFn3.

Enzyme	*V_max_* (U/mg)	*K_m_* (mg/ml)	*k_cat_* (s^−1^)	*k_cat_*/*K_m_* (ml/s/mg)
XynSPP2	226.56	0.97	178.19	183.70
XynSPP2ΔFn3	176.55	1.76	117.27	66.63

### Hydrolytic Properties of XynSPP2

**FIGURE 6:**
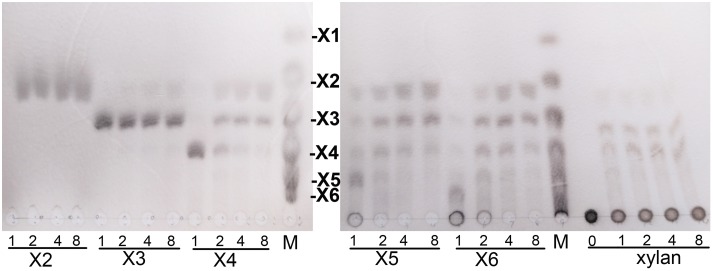
Thin-layer chromatography analysis of XynSPP2-catalyzed products of XOSs and beechwood xylan. XOS standards: X1, xylose; X2, xylobiose; X3, xylotriose; X4, xylotetraose; X5, xylopentaose; and X6, xylohexaose.

Hydrolytic properties of XynSPP2 were investigated with XOSs and beechwood xylan using the whole enzyme XynSPP2. No detectable activity toward xylobiose was observed (Figure 6). Only a small amount of xylotriose was hydrolyzed by XynSPP2 after 8 h of incubation. XynSPP2 hydrolyzed xylotetraose to xylotriose (major) and xylobiose. Degradations of xylopentaose and xylohexaose by XynSPP2 were largely similar, with xylotetraose, xylotriose, and xylobiose as the intermediate enzymatic products and with xylotriose and xylobiose as the final products. The product patterns of beechwood xylan by XynSPP2 after the different incubation periods (1, 2, 4, and 8 h) were nearly similar, with xylotetraose and xylotriose as the major products. No monosaccharide was detected in the enzymatic products of the above substrates (Figure 6).

## Discussion

To date, a large number of GH10 xylanases from bacteria and fungi have been cloned and biochemically characterized (Linares-Pasten et al., 2018). Similar to many other glycoside hydrolases, many xylanases have one or more accessory domains appended to the GH10 catalytic domain through short junction segments (Collins et al., 2005; Talamantes et al., 2016). These auxiliary domains, including carbohydrate-binding modules (CBMs) (Carvalho et al., 2015), dockerin domain (Khan et al., 2013), C-terminal ricin-type β-trefoil lectin domain-like domain (Kim et al., 2018), Fn3 domain (Kim et al., 2009; Chen et al., 2013; Sermsathanaswadi et al., 2017), and S-layer homology domains (Zhao et al., 2013; Lee and Lee, 2014), are non-catalytic, but potentiate the activity of xylanases (Guillén et al., 2010; Sakka et al., 2011) and promote the thermal stability (Kim et al., 2000, 2016) and pH stability (Li et al., 2009; Kim et al., 2016) of the enzymes.

Among the functionally characterized xylanases, only few carry the Fn3 domain (Kim et al., 2009; Chen et al., 2013; Sermsathanaswadi et al., 2017), even though it frequently exists in many other GHs such as cellulases and chitinases. The Fn3 module is important for the catalytic ability of xylanases. Removing the Fn3 domain considerably decreases xylanolytic activity of xylK1 from *Cellulosimicrobium* sp. strain HY-13 (Kim et al., 2009) and Xyn10A from *Flavobacterium johnsoniae* (Chen et al., 2013). However, the specific function of the Fn3 domain in xylanases has yet to be investigated. A previous study proposed that the Fn3 domains in GHs enhance hydrolysis by eroding the surface of large polymeric carbohydrate substrates (Kataeva et al., 2002), being directly involved in binding to the soluble substrate, or being served as linkers synergizing interaction between CBMs and polymeric substrate (Watanabe et al., 1994; Chen et al., 2013). No other accessory domains are fused to XynSPP2 except for the Fn3 domain. Therefore, the Fn3 domain in XynSPP2 likely potentiates the activity of the enzymes by strengthening the binding of the GH10 domain to beechwood xylan, as evidenced by the lower *K_m_* value for XynSPP2 than for XynSPP2ΔFn3.

Amino acid sequence comparison indicated that XynSPP2 is highly identical (84%) to an uncharacterized GH10 xylanase from *L. filiforme* but shares relatively low sequence identity to characterized GH10 xylanases (17–46%). XynSPP2 identified from *Marinifilaceae* bacterium SPP2 was originally isolated from the Antarctic marine sediment (Watanabe et al., 2018). However, the recombinant XynSPP2 showed a temperature optimum of 50°C (Figure 4), which is much higher than the living temperature of its host bacterium (0–25°C) (Watanabe et al., 2018) and the average temperature of Antarctic marine environment (normally approximately 1°C) (Marx et al., 2007). XynSPP2 only showed approximately 20% optimal activity at 20°C and was completely inactive at temperatures below 10°C (Figure 4). The temperature property of XynSPP2 does not resemble those of cold-active enzymes mostly obtained from psychrophilic or psychrotolerant organisms that normally retain large portion of their optimal activities (Santiago et al., 2016). The only feature of XynSPP2 similar to cold-active enzymes is its thermal instability. XynSPP2 is thermolabile because it was unstable at temperatures above 40°C (Figure 4 and Table 2). These temperature properties imply that XynSPP2 has not or incompletely adapted to cold environments during evolution.

**Table 2:** Biochemical characteristics of characterized GH10 xylanases.

Xylanase (source microorganism)	T_*opt*_ pH_*opt*_	pH stability (residual activity)	Thermal stability (residual activity)	Kinetic values (temperature) (substrate)	Hydrolysis products (substrate)	Reference and sequence accession number
XynSPP2 (*Marinifilaceae* bacterium SPP2)	50°C, pH 6	80%, pH 4–11, 1 h	88%, 40°C, 1 h	*K*_*m*_: 0.97 mg/ml, *k*_*cat*_: 178.19 s^−1^ (50°C) (beechwood xylan)	X2, X3, X4 (beechwood xylan)	This work WP_096428726.1
xylK1 (*Cellulosimicrobium* sp. strain HY-13)	55°C, pH 6		50%, 55°C, 20 min		X2, X3, X4 (Birchwood xylan)	Kim et al., 2009 FJ859907
Xyn10A (*Flavobacterium johnsoniae*)	30°C, pH 8	55%, pH 5–9, 1 h	50%, 40°C, 2 h	*K*_*m*_: 5 mg/ml, *k*_*cat*_: 10.7 s^−1^ (35°C) (beechwood xylan)	X1, X2, X3 (beechwood xylan)	Chen et al., 2013 YP_001196196
Xyn10A (*Bacillus* sp. SN5)	40°C, pH 7	80%, pH 5.5–9.9, 24 h	48%, 40°C, 30 min	*K*_*m*_: 0.6 mg/ml, *k*_*cat*_: 85.4 s^−1^ (40°C) (beechwood xylan)		Bai et al., 2012 AGA16736.1
xyl-gt (*Geobacillus thermoleovorans*)	70°C, pH 9	90%, pH 8–10, 3 h	50%, 80°C, 10 min	*K*_*m*_: 0.63 mg/ml, (70°C) (birchwood xylan)	X1, XOS (birchwood xylan)	Verma and Satyanarayana, 2012 AFU93447.1
xynAHJ2 (*Bacillus* sp. HJ2)	35°C, pH 6.5	50%, pH 6–10, 1 h	50%, ≥45°C, 5 min	*K*_*m*_: 0.5 mg/ml, *k*_*cat*_: 11.9 s^−1^ (35°C) (birchwood xylan)		Zhou et al., 2012 AFE82288.1
XynA (*Sorangium cellulosum* So9733-1)	30–35°C, pH 7	60%, pH 6–9, 1 h	20%, 50°C, 20 min	*K*_*m*_: 25.8 mg/ml, *k*_*cat*_: 6.8 s^−1^ (30°C) (beechwood xylan)	X1, X2 (beechwood xylan)	Wang et al., 2012 AEB69780.1
XylC (*Cohnella laeviribosi* HY-21)	50°C, pH 7.5		50%, 50°C, 15 min			Kim et al., 2010 EDV78425
XynA (*Glaciecola mesophila* KMM241)	30°C, pH 7	80%, pH 6–8, 1 h	20%, 30°C, 1 h	*K*_*m*_: 1.22 mg/ml, *k*_*cat*_: 69 s^−1^ (30°C) (beechwood xylan)	X2, X3 (beechwood xylan)	Guo et al., 2009 ACN76857.1

The activity of XynSPP2 was suppressed in the presence of many divalent cations (Co^2+^, Ca^2+^, Fe^2+^, Mg^2+^, Mn^2+^, Ni^2+^, and Zn^2+^) at a concentration of 5 mM (Figure 5). Similar results were observed in Xyn10A from *Bacillus* sp. SN5 (Bai et al., 2012) and xyl-gt from *Geobacillus thermoleovorans* (Verma and Satyanarayana, 2012; Table 2). By contrast, the xylanase activities of many GH10 xylanases can be improved by adding a certain metal ion, such as Xyn10A from *F. johnsoniae* by Mn^2+^ (Chen et al., 2013), xylK1 (Kim et al., 2009) by Fe^2+^, xynAHJ2 from *Bacillus* sp. HJ2 by Mg^2+^ (Zhou et al., 2012), XynA from *Sorangium cellulosum* (Wang et al., 2012) by Ca^2+^ and Mg^2+^, and XylC from *Cohnella laeviribosi* HY-21 by Ni^2+^ and Mn^2+^ (Kim et al., 2010). The activity of XynSPP2 was inhibited by EDTA (5 mM). Similar adverse effects of EDTA have been reported for the most tested GH10 xylanases, including XylC (*C. laeviribosi* HY-21) (Kim et al., 2010), xyl-gt (Verma and Satyanarayana, 2012), Xyn10A from *Bacillus* sp. SN5 (Bai et al., 2012), XynA from *S. cellulosum* (Wang et al., 2012), Xyn10A from *F. johnsoniae* (Chen et al., 2013), and XylK1 (Kim et al., 2009). The positive effects of EDTA on GH10 xylanases have been observed for XynA from *Glaciecola mesophila* KMM 241 (Guo et al., 2009) and xynAHJ2 from *Bacillus* sp. (Zhou et al., 2012). Reducing reagents DTT and β-mercaptoethanol (both at 10 mM) suppressed the activity of XynSPP2 by 15–20%, similar to that observed for xyl-gt (Verma and Satyanarayana, 2012). Anionic detergent SDS normally showed inhibitory effects on xylanase activity. In the presence of SDS at 10 mM, the activity of XynSPP2 reduced by 45%. XynSPP2 was more susceptible to SDS than Xyn10A from *Bacillus* sp. SN5 (5 mM, 55% residual activity) (Bai et al., 2012) and Xyn10A from *F. johnsoniae* (17 mM, 64% residual activity) (Chen et al., 2013), but the SDS tolerance of XynSPP2 was much higher than that of XynA from *S. cellulosum* (10 mM, 0.5% residual activity) (Wang et al., 2012) and XynA from *G. mesophila* KMM 241 (10 mM, 0% residual activity) (Guo et al., 2009). Non-ionic surfactant Triton-X 100 (0.5%) improved the activity of XynSPP2 by 13%. The activity promotion of XynSPP2 by Triton X-100 was comparable to that of Xyn10A from *F. johnsoniae* (0.5%, 114% residual activity) (Chen et al., 2013) but was weaker than that of XylC from *C. laeviribosi* HY-21 (0.5%, 222% residual activity) (Kim et al., 2010) and XylK1 (0.5%, 180% residual activity) (Kim et al., 2009). XynSPP2 was more susceptible to the tested organic solvents at 5%, compared with xyl-gt (Verma and Satyanarayana, 2012), Xyn10A from *Bacillus* sp. SN5 (Bai et al., 2012), and Xyn10A from *F. johnsoniae* (Chen et al., 2013).

**FIGURE 7:**
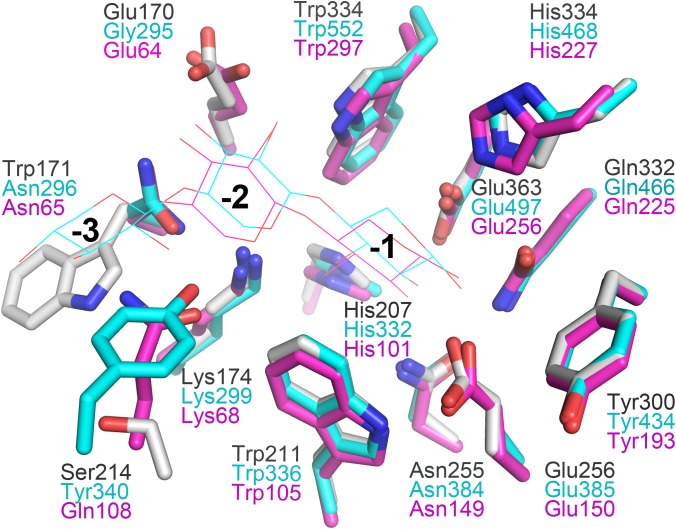
Superimposition of the subsites of XynSPP2 (gray), *Cj*Xyn10C (xylanase from *Cellvibrio japonicas* in complex with xylotriose, PDB entry:1US2, cyan), and *Tp*Xyl10B (xylanase from *Thermotoga petrophila RKU-1* in complex with xylobiose, PDB entry: 3NJ3, magenta). The amino acid and xylose residues are shown in stick and line models, respectively.

In general, the hydrolytic product patterns of xylanases are dependent on the interactions between subsites and xylose residues in the active-site cleft. Without considering the effect of heteroxylan structure, the subsite topology and composition determine the positional binding and cleavage (Schmidt et al., 1999; Pollet et al., 2010; Linares-Pasten et al., 2018). Structural studies have shown that the glycone subsites of GH10 xylanases are highly conserved on the whole and crucial for substrate binding (Pollet et al., 2010). Structural comparison of XynSPP2 with other GH10 xylanases revealed that XynSPP2 had an unusual amino acid composition at glycone subsites (Figure 1, 2, 7). Two conserved amino acid residues (asparagine and glutamine), as the constituent part of the −2/3 subsite (in the distal region of −2 subsite (−2/3) and form important hydrogen bonds with O3 and O5 in −2 xylose residue) of the GH10 xylanases, are substituted by a tryptophan and an asparagine in XynSPP2 (Figure 7). It has been for example showed that natural variation (Glu/Gly substitution) (Figure 7) at the −2/−3 subsite of *Cj*Xyn10C weakens the affinity for XOSs (X3–X6) (10–100-fold lower) but not xylan (Pell et al., 2004). Another variation at the −2/−3 subsite of *Cj*Xyn10C changes the cleavage pattern of the enzyme (Pell et al., 2004). Inserting tyrosine residue (Tyr340) at the −2/−3 subsite established a strong −3 subsite through forming stacking interaction with −3 xylose residue (Figure 7). With this tyrosine residue, the enzyme becomes more inclined to cleave the terminal glyosidic bond of xylotetraose rather than to act in a symmetric cleavage manner (Pell et al., 2004). Trp171, one of the two variant amino acids in the active-site cleft of XynSPP2, has similar position and biochemical property to Tyr340. Therefore, we speculate that aromatic Trp171 in XynSPP2 possibly forms a strong −3 subsite for xylose binding as Tyr340 in *Cj*Xyn10C (Pell et al., 2004). Accordingly, a similar substrate-binding mode as for *Cj*Xyn10C may be adopted by XynSPP2.

Due to the strong and specific binding at the glycone subsites (−2 to −1 or −3 to −1 when −3 subsite exists), the number of strong glycone subsites in the active-site cleft has been proposed to be the determinant for the minimum size of XOSs as substrates for effective hydrolysis (Schmidt et al., 1999; Linares-Pasten et al., 2018; Nordberg Karlsson et al., 2018). Therefore, the presence of a putative strong −3 subsite in XynSPP2 suggested that XOSs longer than xylotriose can be effectively cleaved by the enzyme, as evidenced by our TLC experiment. The TLC results indicated that the shortest substrate that was effectively cleaved by XynSPP2 is xylotetraose (Figure 6). With xylotetraose as the substrate, xylotriose predominantly appeared in the intermediate sample (2 h), suggesting that xylotetraose was more frequently cleaved into xylotriose and xylose, although a −2 to +2 cleavage also occurred. Digestion of xylopentaose yielded mainly xylotriose and xylobiose. According to the architecture of the glycone subsites in XynSPP2 and the product patterns of XOSs, we can speculate that the enzyme had at least four subsites to be filled for substantial activity. Xylotetraose mainly occupied subsites −3 to +1 and xylopentaose predominantly bound at −3 to +2 subsites. Short XOSs (xylobiose and xylotriose) were not easily hydrolyzed by XynSPP2 because the whole molecules bound to the glycone subsites. Notably, no detectable xylose was observed in all hydrolysis samples which was probably ascribed to a transxylosylation reaction catalyzed by XynSPP2. The released xylose molecules were transferred to the covalent xylosyl-enzyme intermediate, generating long XOSs such as xylotetraose or xylopentaose. The lack of xylose in the degradation product of XOSs was also observed for xylanases XylK1 and XylK2 from *Cellulosimicrobium* sp. strain HY-13, both of which displayed apparent transxylosylation activity (Kim et al., 2009, 2012).

## Conclusion

In this study, a new bimodular xylanase, XynSPP2 from *Marinifilaceae* bacterium strain SPP2, was expressed and characterized. It is the first xylanase characterized from the family *Marinifilaceae*. XynSPP2 showed maximum activity at 50°C and pH 6 and was stable over a broad pH range and temperature lower than 50°C. XynSPP2 displays several characteristics compared to many other characterized GH10 xylanases. First, the enzyme shares very low amino acid identity to those characterized GH10 xylanases (17–46%). Second, it is a rare Fn3-fused xylanase and the Fn3 domain is beneficial to the activity of the enzyme. Third, it has a unique −2/−3 subsite which updates our understanding of “polymorphisms” in the substrate binding cleft of GH10 xylanases. An Asn/Try substitution renders a strong −3 subsite in XynSPP2 which results in low activity of the enzyme toward short XOS (xylotriose). Fourth, with xylan as substrate, no detected monosaccharide is produced due to putative transxylosylation activity of XynSPP2. The substrate preference for long XOSs (>xylotriose) and the lack of monosaccharide production to a certain extent can be an advantage for XynSPP2 in prebiotic XOS production.

## Data Availability

Publicly available datasets were analyzed in this study. This data can be found here: https://www.ncbi.nlm.nih.gov/nuccore/NZ_AP018042.1.

## Author Contributions

JY designed the experiments. ZH and FS-g conducted the experiments and analyzed the data. ZH wrote the manuscript.

## Conflict of Interest Statement

The authors declare that the research was conducted in the absence of any commercial or financial relationships that could be construed as a potential conflict of interest.

## Footnotes

1 http://www.cazy.org/

2 http://www.cbs.dtu.dk/services/SignalP/

3 https://www.ncbi.nlm.nih.gov/Structure/cdd/wrpsb.cgi

4 https://blast.ncbi.nlm.nih.gov/Blast.cgi

5 http://www.cazy.org/GH10_characterized.html

6 https://www.ebi.ac.uk/Tools/msa/clustalo/

7 http://swissmodel.expasy.org/
