# Supplementary material for: Molecular and Biochemical Characterization of a Bimodular Xylanase From Marinifilaceae Bacterium Strain SPP2
Source: Front Microbiol. 2019 Jul 2;10:1507. doi: 10.3389/fmicb.2019.01507 (PMC6614494; doi:10.3389/fmicb.2019.01507)
Supplement: Supplementary file 1 [file Data_Sheet_1.PDF]

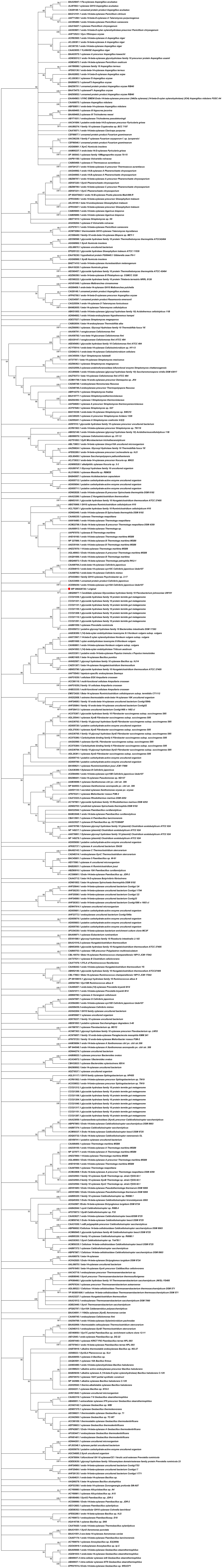

Figure S1. Evolutionary relationships between XynSPP2 and 320 amino acid sequences of characterized GH10 xylanases. The analysis Evolutionary analyses were conducted in MEGA7. The phylogenetic tree was constructed using the Neighbor-Joining method. The xylanases are represented by their relevant GenBank accession numbers. The number of times each branch is shown as a percentage. The percentage (shown next to the branches) of replicate trees was supported from 1000 bootstraps.
