# Supplementary material for: Molecular and Biochemical Characterization of a Bimodular Xylanase From Marinifilaceae Bacterium Strain SPP2
Source: Front Microbiol. 2019 Jul 2;10:1507. doi: 10.3389/fmicb.2019.01507 (PMC6614494; doi:10.3389/fmicb.2019.01507)
Supplement: Supplementary file 2 [file Data_Sheet_2.PDF]

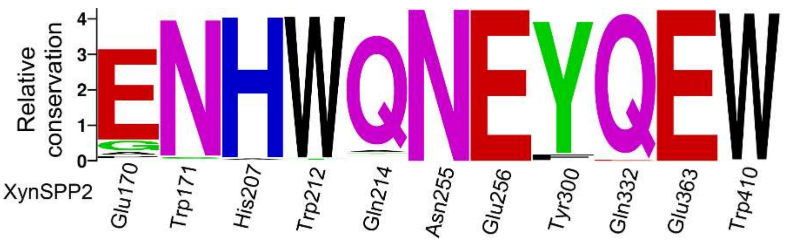

Figure S2. Amino acids in the active-site cleft of GH10 xylanases and their conservations. The size of letter indicates the conservative degree of the give position. The corresponding amino acid residues in XynSPP2 are shown below.
