# Supplementary material for: Molecular and Biochemical Characterization of a Bimodular Xylanase From Marinifilaceae Bacterium Strain SPP2
Source: Front Microbiol. 2019 Jul 2;10:1507. doi: 10.3389/fmicb.2019.01507 (PMC6614494; doi:10.3389/fmicb.2019.01507)
Supplement: Supplementary file 3 [file Data_Sheet_3.PDF]

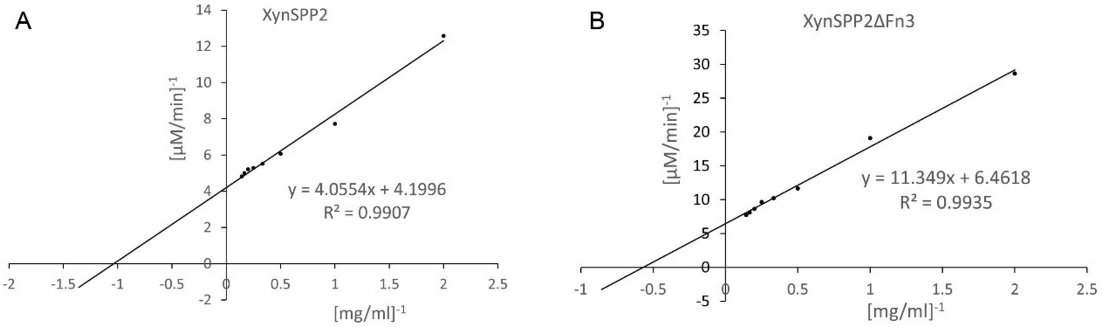

Figure S3. Reciprocal Lineweaver-Burk plots used determine the kinetic parameters of XynSPP2 (**A**) and XynSPP2△Fn3 (**B**) with beechwood xylan as substrate. All data shown are mean values from at least three replicate experiments.
